# Feature selection with a genetic algorithm can help improve the distinguishing power of microbiota information in monozygotic twins' identification

**DOI:** 10.3389/fmicb.2023.1210638

**Published:** 2023-07-24

**Authors:** Guangping Fu, Guanju Ma, Shujie Dou, Qian Wang, Lihong Fu, Xiaojing Zhang, Chaolong Lu, Bin Cong, Shujin Li

**Affiliations:** ^1^College of Forensic Medicine, Hebei Medical University, Hebei Key Laboratory of Forensic Medicine, Research Unit of Digestive Tract Microecosystem Pharmacology and Toxicology, Chinese Academy of Medical Sciences, Shijiazhuang, China; ^2^Hainan Tropical Forensic Medicine Academician Workstation, Haikou, China

**Keywords:** forensic microbiology, monozygotic twins, personal identification, 16s rRNA sequencing, machine learning, genetic algorithm

## Abstract

**Introduction:**

Personal identification of monozygotic twins (MZT) has been challenging in forensic genetics. Previous research has demonstrated that microbial markers have potential value due to their specificity and long-term stability. However, those studies would use the complete information of detected microbial communities, and low-value species would limit the performance of previous models.

**Methods:**

To address this issue, we collected 80 saliva samples from 10 pairs of MZTs at four different time points and used 16s rRNA V3–V4 region sequencing to obtain microbiota information. The data formed 280 inner-individual (Self) or MZT sample pairs, divided into four groups based on the individual relationship and time interval, and then randomly divided into training and testing sets with an 8:2 ratio. We built 12 identification models based on the time interval ( ≤ 1 year or ≥ 2 months), data basis (Amplicon sequence variants, ASVs or Operational taxonomic unit, OTUs), and distance parameter selection (Jaccard distance, Bray-Curist distance, or Hellinger distance) and then improved their identification power through genetic algorithm processes. The best combination of databases with distance parameters was selected as the final model for the two types of time intervals. Bayes theory was introduced to provide a numerical indicator of the evidence's effectiveness in practical cases.

**Results:**

From the 80 saliva samples, 369 OTUs and 1130 ASVs were detected. After the feature selection process, ASV-Jaccard distance models were selected as the final models for the two types of time intervals. For short interval samples, the final model can completely distinguish MZT pairs from Self ones in both training and test sets.

**Discussion:**

Our findings support the microbiota solution to the challenging MZT identification problem and highlight the importance of feature selection in improving model performance.

## 1. Introduction

Distinguishing monozygotic twins (MZT) has long been a challenging task in individual identification, and it has significant implications in some cases. For example, Jobling (2013) introduced several of such cases, including a rape case where the characterization was obstructed by the existence of the suspect's MZT brother and the difficulty of distinguishing them. Various techniques have been established to identify MZTs. One such technique involves the analysis of Single Nucleotide Polymorphism (SNP), Count Number Variation (CNV), and Insertion/Deletion variation (InDel) markers (Hannelius et al., 2007; Abdellaoui et al., 2015; Xu et al., 2017). Researchers have reported that there may be differences in these genomic biomarkers between MZTs. However, due to the nearly identical nuclear and mitochondrial genome information shared by MZTs, such differences are infrequent (McRae et al., 2015; Ming et al., 2019) and genome-wide sequencing is typically necessary to differentiate MZTs. Another MZT identification strategy involves the investigation of various biomarkers from the perspective of epigenetics, including DNA methylation (Stewart et al., 2015; Xu et al., 2015) and microRNA (Fang et al., 2019; Xiao et al., 2019). Applying such biomarkers, the difference between MZTs would be more significant and the identification efficacy can be promising (Xu et al., 2015). However, using such biomarkers would necessitate strict material standards which would limit their application in daily work. Specially, RNA is easily degraded by exonuclease hydrolysis in the environment and DNA methylation method requires high quality and quantity of DNA because sodium bisulfite may lead to DNA fragmentation and loss. Therefore, more suitable biomarkers for MZT identification are still required.

Advances in microbiology may provide another possible solution to the MZT identification problem. As it is known, human bodies contain two genomes: the human genome is inherited from parents, and the microbiota reside on/in the human body. The latter, known as the “second genome” of the human body, is thought to contain genetic information that is ~50–100 times greater than the human genome (Grice and Segre, 2012). Recent advances in sequencing technology have helped the growth of forensic microbiology, an interdisciplinary field of forensic medicine and microbiology (Ventura Spagnolo et al., 2019; Speruda et al., 2022). With a sharp focus much attention has been focused on the application of microbial information, with a particular emphasis on postmortem interval estimation (Liu et al., 2022), cause of death inference (Kaszubinski et al., 2020), body fluid type identification (Yao et al., 2021), and individual identification (Watanabe et al., 2018).

Numerous studies have revealed that everyone has a unique microbiome and differs, including between MZTs (Stahringer et al., 2012; Abeles et al., 2014; Suzuki et al., 2022; Sukumar et al., 2023). For example, Stahringer et al. (2012) demonstrated that the difference in salivary microbiome 16s rRNA data between MZT individuals was not less than that between dizygotic twins (DZT) and that this difference gradually increased after they lived apart. Based on such differences, primary studies on the MZT identification model constructions have been conducted. For example, Bozza et al. (2022) collected saliva samples from individuals with different relationships (unrelated individuals, MZT, or inner-individual samples) for sequencing and then built a model that can roughly distinguish between MZT and inner-individual samples. However, such studies would include all tested species in the model construction. In theory, the sensitivity of different microbial species to environmental changes should differ, resulting in different stability within the same individual. Therefore, changes in sequencing data of relatively unstable species would partially offset support for the same identification from relatively stable species. Therefore, there is still room for improvement in this field.

In this study, 80 saliva samples were collected from 10 MZT pairs to collect microbiota information via 16s rRNA sequencing. Twelve models were developed, each considering sample collection intervals, sequence data basis, and distance selection. High-value species were selected using Genetic Algorithm (GA) processes in such models. Finally, the two models with the best distinguishing power in the training test (under two different sampling intervals) were selected. The model developed for the short interval samples could completely separate MZT from inner-individual sample pairs in training and test sets.

## 2. Materials and methods

### 2.1. Sample collection

The present study included 10 pairs of MZT (four male and six female pairs). Participants signed informed consent forms before data collection, and the research content met the medical ethics requirements of Hebei Medical University. For each participant, four freely flowing saliva samples (1 mL each) were collected at four different time points (TP 1-4), the second, third, and fourth of which were collected 12, 13, and 14 months after the first one. Each participant stated that they had not used antibiotics within 6 months before sampling and had not consumed food or water within 2 h of sampling. A total of 80 samples were collected and labeled based on the participant and the time point. For example, “S10A1” denoted the sample belonging to individual A of the 10th MZT pair and was collected at the first time point. Genomic DNA was extracted from the saliva samples using the TGuide S96 Magnetic Soil/Stool DNA Kit (TIANGEN Biotech, Beijing) as recommended by the manufacturer and stored at −80°C until needed.

### 2.2. Library preparation and sequencing

A two-round-PCR workflow was used for the library preparation for the 16s rRNA V3–V4 sequencing that involves four steps: **(i) First round PCR:** The following primers were used to amplify the target regions of 16S rDNA: 338F: 5′-ACTCCTACGGGAGGCAGCA-3′ and 806R: 5′-GGACTACHVGGGTWTCTAAT-3′. PCR was performed in 10 μL reactions, with 0.3 μL forward primers (10 μM), 0.3 μL reverse ones (10 μM), 50 ng ± 20% template DNA, 0.2 μL KOD FX Neo, 5 μL KOD FX Neo Buffer, and 2 μL dNTP (2 mM of each type). The PCR program consisted of 95°C for 5 min, followed by 25 cycles of 95°C for 30 s, 50°C for 30 s, and 72°C for 40 s, with a final extension at 72°C for 7 min. **(ii) Second round PCR:** The first round PCR product was used as a template in the second round PCR to add index labels to each sample. PCR was performed in 20 μL reactions containing 5 μL of the first PCR round product, 2.5 μL MPPI-a (2 μM), 2.5 μL MPPI-b (2 μM), and 10 μL 2 × Q5 HF MM. The PCR program consisted of 98°C for 30 s, followed by 10 cycles of 98°C for 10 s, 65°C for 30 s, and 72°C for 30 s, with a final extension at 72°C for 5 min. **(iii) Quantifying and sample mixing:** The integrity of the second round PCR product for each sample was tested using 1.8% agarose gel electrophoresis (120 V, 40 min), and then quantitative analysis was performed based on electrophoresis findings using ImageJ software. The samples were mixed by equal mass based on the quantification results. **(iv) Purifying and recovering:** The mixed samples were purified using OMEGA DNA purification columns. The fragments with target lengths were then cut and recovered in a second round of 1.8% agarose gel electrophoresis (120 V, 40 min).

Then, sequencing was performed on the Illumina Novaseq 6000 sequencing platform using a double-end sequencing strategy (PE250, Biomarker Technologies Co, Ltd., Beijing, China) according to standard protocols.

### 2.3. Preliminary analysis of microbial diversity

Several steps were taken on the original sequence data to obtain high-quality reads: (i) FLASH (Magoč and Salzberg, 2011) (version 1.2.11) was used to splice the original sequencing data and obtain Raw Tags, with a minimum overlap length of 10 bp and a maximum mismatch ratio set as 0.2; (ii) Trimmomatic (Bolger et al., 2014) (version 0.33) was used to filter the spliced sequence and obtain the Clean Tags: Raw Tags were checked sequentially using a 50 bp window, once the average quality value within the window was <20, the subsequent bases would be truncated. Truncated Tags with <75% length would be filtered; (iii) UCHIME (Edgar et al., 2011) (version 8.1) was used to obtain Clean Reads by removing chimeras. The threshold for confirming a chimera was set at 80% similarity between the query sequence and parent sequences selected from the reference database.

Two strategies were employed to achieve two types of microbial information (i.e., the “feature” to be selected) based on these filtered high-quality sequences: (i) The UPARSE algorithm was used on USEARCH software version 10.0 (Edgar, 2013) to cluster the sequences at a level of 97% similarity to obtain Operational Taxonomic Unit (OTU); and (ii) the dada2 workflow (Callahan et al., 2016) on QIIME2 platform (Bolyen et al., 2019) (2020.6) was used to perform noise reduction and to obtain Amplicon Sequence Variant (ASV, i.e., OTU with a level of 100% similarity). The OTUs/ASVs with <0.005% of total reads were filtered out (Bokulich et al., 2013). The SILVA database Release 138.1 (Quast et al., 2012) was used as a reference for species annotation based on ASV information, using a naive Bayesian classifier combined with alignment. RDP Classifier version 2.2 (Wang et al., 2007) was used as the annotating tool. The abundance information of various species at different taxonomic levels was then summarized using QIIME2.

The Chao 1, ACE, Shannon-wiener, and Simpson indices were calculated using Mothur v.1.30 (Schloss et al., 2009) for alpha diversity analysis for each sample. In addition, principal coordinate analysis (PCoA) was performed using the distance matrix of beta diversity parameters (such as Bray-Curtis distance).

### 2.4. Feature selection to promote the distinguishing ability of microbial model in MZT identification

#### 2.4.1. Division of data sets

There would be eight samples for each MZT pair, resulting in $C_{8}^{2}=28$ sample pairs. All the 280 pairs (28 sample pairs per MZT pair × 10 MZT pairs) were divided into four groups based on two dimensions: (i) the relationship between the individuals from which the two samples were collected (samples from the same individual were labeled as “Self,” and samples from different individuals within an MZT pair were labeled as “MZT”); and (ii) the sample collection interval ( ≤ 2 months was labeled as “short”, or ≥ 12 months was labeled as “long”). The sample pairs in the four groups were randomly divided into training or test sets with an 8:2 ratio, where the test set is used only for the validation of the final models. The sample amounts in each group are listed in Table 1.

**Table 1:** The division of data sets.

**Relationship**	**Interval**	**Training set**	**Test set**
Self	Short	48	12
	Long	48	12
MZT	Short	80	20
	Long	48	12

#### 2.4.2. Feature type and beta distance parameter choice

As stated in Section 2.3, two types of features (i.e., microbial sequencing information) would be used as the data basis for model construction: OTUs or ASVs. Three types of beta diversity distances were calculated based on these two types of features:

**(i) Jaccard distance (JD)**, which only considers whether a specific feature was detected in two samples. JD between two samples, A and B, is denoted as *JD*(*A, B*) and calculated as shown in Equation (1), if the symbol “|*A*∩*B*|” denoted the number of features detected in both samples A and B, and “|*A*∪*B*|” represented the total number of features detected in the two samples.


(1) $$\begin{array}{l} JD\left(A,B\right)=1-\frac{\left|A\cap B\right|}{\left|A\cup B\right|} \end{array}$$

**(ii) Bray-Curtis Distance (BC)**, which represents the difference in absolute abundance information between two samples of the tested features, calculated as Equation (2), if symbols “*a*_*i*_” and “*b*_*i*_” denote the absolute abundance of the *i*th feature in samples A and B, respectively.


(2) $$BC\left(A,B\right)=\frac{\sum_{i}\left|a_{i}-b_{i}\right|}{\sum_{i}\left(a_{i}+b_{i}\right)}$$

**(iii) Hellinger distance (HD)**, which calculates the difference in relative abundance information between two samples of the tested features, calculated as Equation (3), if “$\underset{j}{\sum }a_{j}$” and “$\underset{j}{\sum }b_{j}$” denote the total abundance of all features considered in samples A and B, respectively.


(3) $$HD\left(A,B\right)=\frac{1}{\sqrt{2}}\sqrt{\sum_{i}{\left(\sqrt{\frac{a_{i}}{\sum_{j}a_{i}}}-\sqrt{\frac{b_{i}}{\sum_{j}b_{i}}}\right)}^{2}}$$

In summary, six possible models could be constructed based on data basis and distance choices for distinguishing the Self and MZT pairs under each sampling interval. Therefore, a total of 12 models will be constructed. The distinguishing ability of the 12 choices would be measured based on all features detected. The area under the receiver operating characteristic curve (AUC) would be used in such measurements. The AUC parameter serves as a commonly used metric for evaluating binary classifiers. For each of the 12 models, the following steps can be taken to calculate the AUC: Firstly, calculation results for all sample pairs are sorted from smallest to largest and assigned rank numbers, during which the average rank would be assigned for pairs with identical results. Secondly, if the number of MZT pairs and Self pairs are indicated as *n*_*M*_ and *n*_*S*_, respectively, and the sum of ranks in MZT pairs is represented with symbol “*R*_*M*_,” the AUC can be obtained by using the following equation:


(4) $$\begin{array}{l} AUC=\frac{R_{M}-\frac{n_{M}\left(n_{M}+1\right)}{2}}{n_{M}\times n_{S}} \end{array}$$

#### 2.4.3. Feature selection based on genetic algorithm

To construct more efficient discrimination models, GAs were performed 12 times on MATLAB (R2021a update 4, MathWorks, United States) to screen the high-value OTUs or ASVs in the identification models. Since their introduction in the 1970s, GAs have been widely used in feature selection. The algorithm uses mathematical methods and computer simulation operations to transform the process of solving complex problems into processes similar to the chromosome genes crossover and mutation in biological evolution. Then it searches for the optimal solution by simulating natural evolution processes.

The operational process of GA involves several fundamental concepts that are presented in bold and italicized words: (i) An ***individual*** presents a potential solution to the problem. Each ***individual*** is a digital string of the same length as the number of detected features in all samples. Each position on the string is called a ***gene***, one-to-one corresponding to the detected features. Each ***gene*** has two possible states called ***alleles***, 1 if the corresponding feature is selected or 0 if not. (ii) A ***population*** is a group of ***individuals***. Besides the initial ***population*** [**P(0)**], ***populations*** would be generated iteratively and denoted as **P(*x*)**, where “***x***” denote the iteration generation and ***x*** = 10,000 is set as the first termination condition. (iii) The value of an ***individual*** in MZT identification would be measured with the “fitness function.” In the present study, the corresponding distance parameter would be calculated for each pair in the training set with the corresponding interval based on the features being selected according to a specific ***individual*** (i.e., the corresponding ***allele*** being 1); then AUC would be calculated with Equation (4) and used as the fitness function. The second termination condition would be AUC = 1, indicating that the two groups can be completely separated.

The GA operation would be carried out in the following steps for each of the 12 model construction processes:

**(i) Initialization:** Randomly generate 2,000 ***individuals*** to form **P(0)**. Calculate AUC for each ***individual***; if the second termination condition is fulfilled, output the optimal solution directly; otherwise, enter the evolution process.

**(ii) Evolution:** Three steps would be taken to generate a new ***population***, **P(*x*+1)**, from the existing one, **P(*x*)**:

**(a) Selection:** ***Individuals*** with the lowest 10% AUC in **P(*x*)** would be eliminated. According to their AUCs, 2,000 “fathers” and 2,000 “mothers” would be selected from the remaining 90% **P(*x*)** ***individuals*** according to their AUC:


(5) $$\begin{array}{l} Pr\left(individual\;i\;is\;selected\right)=\frac{{AUC}_{i}}{\underset{j\in the\;top\;90\%\;individuals}{\sum }{AUC}_{j}} \end{array}$$

**(b) Crossover:** ***Individuals*** in **P(*x*+1)** would inherit ***alleles*** from their “parents,” ***alleles*** from both of which have the same probability of being inherited for each gene.

**(c) Mutation:** A part of the ***alleles*** in the next generation would be reversed between 0 and 1. In the present study, the mutation rate, i.e., the probability of such reversion occurring, was 1%.

**(iii) Evaluation and iteration:** Calculate AUC for each ***individual*** of **P(*x*+1)**. If any of the two termination conditions are fulfilled, output the optimal solution and terminate the process; otherwise, repeat the evolution process in step (ii).

After the 12 GA processes, select the two feature-distance combinations with the best AUCs for each type of interval to build the MZT identification model.

### 2.5. Introducing Bayes theory in the final models

After feature selection, the specific distance between a sample pair can be used to infer the relationship between the sourcing individuals. However, in some cases, it may be necessary to answer the question, “How confident are we in judging the relationship between these two individuals.” The likelihood ratio calculation could be helpful in answering the question.

#### 2.5.1. Basic theories

When only one individual of the MZT pairs is available as the suspect for MZT identification, there are more than two exclusive assumptions (Bozza et al., 2022), such as *H*_1_: the query saliva trace originates from the suspect; or *H*_2_: the query saliva trace originates from the suspect's MZT sibling. The present study aimed to estimate the probability of *H*_1_ being true, considering the distance obtained by sequencing and calculating, i.e., *Pr*(*H*_1_|*D* = *d*) where “*d*” stands for the specific distance value and “*D* = *d*” denote the event that *d* is calculated between the two samples. According to the Bayes Rules, such a probability can be calculated by Equation (6):


(6) $$\begin{array}{l} Pr\left(H_{1}|D=d\right)=\frac{Pr\left(H_{1}\right)\times Pr\left(D=d|H_{1}\right)}{Pr\left(D=d\right)} \end{array}$$

Where *Pr*(*H*_1_) denote the probability of *H*_1_ being true without considering the microbial evidence, and *Pr*(*D* = *d*) denote the probability of two individuals exhibiting corresponding distance. *Pr*(*D* = *d*) is usually difficult to calculate accurately because there may be infinite hypotheses, resulting in a high difficulty in accurately calculating *Pr*(*H*_1_|*D* = *d*). However, calculating the ratio of it to the probability of another hypothesis being true would be much easier, such as:


(7) $$\begin{array}{l} \frac{Pr\left(H_{1}|D=d\right)}{Pr\left(H_{2}|D=d\right)}=\frac{Pr\left(H_{1}\right)}{Pr\left(H_{2}\right)}\times \frac{Pr\left(D=d|H_{1}\right)}{Pr\left(D=d|H_{2}\right)} \end{array}$$

Where the ratio *Pr*(*H*_1_)/*Pr*(*H*_2_) is known as the ratio of prior probabilities, and if the probability of any hypothesis being true is assumed to be equal, such a ratio should equal 1. Thus, if we define a parameter likelihood ratio (LR or BF in some studies; Bozza et al., 2022) as Equation (8), it can represent the degree to which *H*_1_ is more likely to be true than *H*_2_. When considering a determined value, the probability ratio should equal the ratio between the probability density in the Self group and that in the MZT group, labeled with $\hat{f}$:


(8) $$\begin{array}{l} LR=\frac{Pr\left(D=d|H_{1}\right)}{Pr\left(D=d|H_{2}\right)}=\frac{\hat{f}\left(d|Self,short/long\right)}{\hat{f}\left(d|MZT,short/long\right)} \end{array}$$

#### 2.5.2. Model construction based on LR results

To obtain the two probabilities in Equation (8), the distribution of the specific distance between Self or MZT sample pairs must be estimated while considering the time interval. Gaussian Kernel density estimation (KDE) was used as the estimation method. Assume that n pairs of specific samples are tested, and the specific distance between each pair is labeled as *X*_1_, *X*_2_, ..., and *X*_*n*_, then the probability density can be estimated by Equation (9):


(9) $$\begin{array}{l} \hat{f}\left(d\right)=\frac{1}{nh\sqrt{2\pi }}\overset{n}{\underset{i=1}{\sum }}\left[e^{-\frac{{\left(d-X_{i}\right)}^{2}}{2h^{2}}}\right] \end{array}$$

And the bandwidth *h* was set as Equation (10), where δ denotes the standard deviation of all *X*_*i*_.


(10) $$\begin{array}{l} h=1.06\delta n^{-\frac{1}{5}} \end{array}$$

Thus, the LR of each pair could be calculated by Equation (11)


(11) $$\begin{array}{l} LR=\frac{n_{M}h_{M}\overset{n_{S}}{\underset{i=1}{\sum }}\left[e^{-\frac{{\left(d-S_{i}\right)}^{2}}{2h_{S}^{2}}}\right]}{n_{S}h_{S}\overset{n_{M}}{\underset{j=1}{\sum }}\left[e^{-\frac{{\left(d-M_{j}\right)}^{2}}{2h_{M}^{2}}}\right]} \end{array}$$

Where *n*_*M*_ or *n*_*S*_ denotes the number of MZT or Self pairs within a specific time interval, and *h*_*M*_ or *h*_*S*_ represents the bandwidth of corresponding groups. *S*_*i*_ denotes the specific distance between the *ith* Self pair and *M*_*j*_ the distance between the *jth* MZT pair.

After calculating LR for each of the 224 sample pairs, diagnostic tests were performed between MZT and Self pairs at each time interval. Two types of thresholds would be used in the final models: (i) *T*_*max*_: the LR thresholds that may provide the best Youden index [YI, which can be calculated with Equation (12), if *Sen* and *Spe* denote sensitivity and specificity, respectively] in the training sets; and (ii) LR = 1.


(12) $$\begin{array}{r} \begin{array}{l} Sen=\frac{number\;of\;Self\;pairs\;being\;confirmed\;as\;Self\;pairs}{n_{S}}\\ Spe=\frac{number\;of\;MZT\;pairs\;being\;confirmed\;as\;MZT\;pairs}{n_{M}} \end{array}\}\\ \Rightarrow YI=Sen+Spe-1 \end{array}$$

### 2.6. Validation of the model

Based on the two feature-distance combinations selected, LRs would be calculated for each of the 56 sample pairs in the test set, and diagnostic tests would be performed. The validation parameter of the final models would be YI under the two types of threshold sets.

## 3. Results

### 3.1. Sequencing results

**Figure 1:**
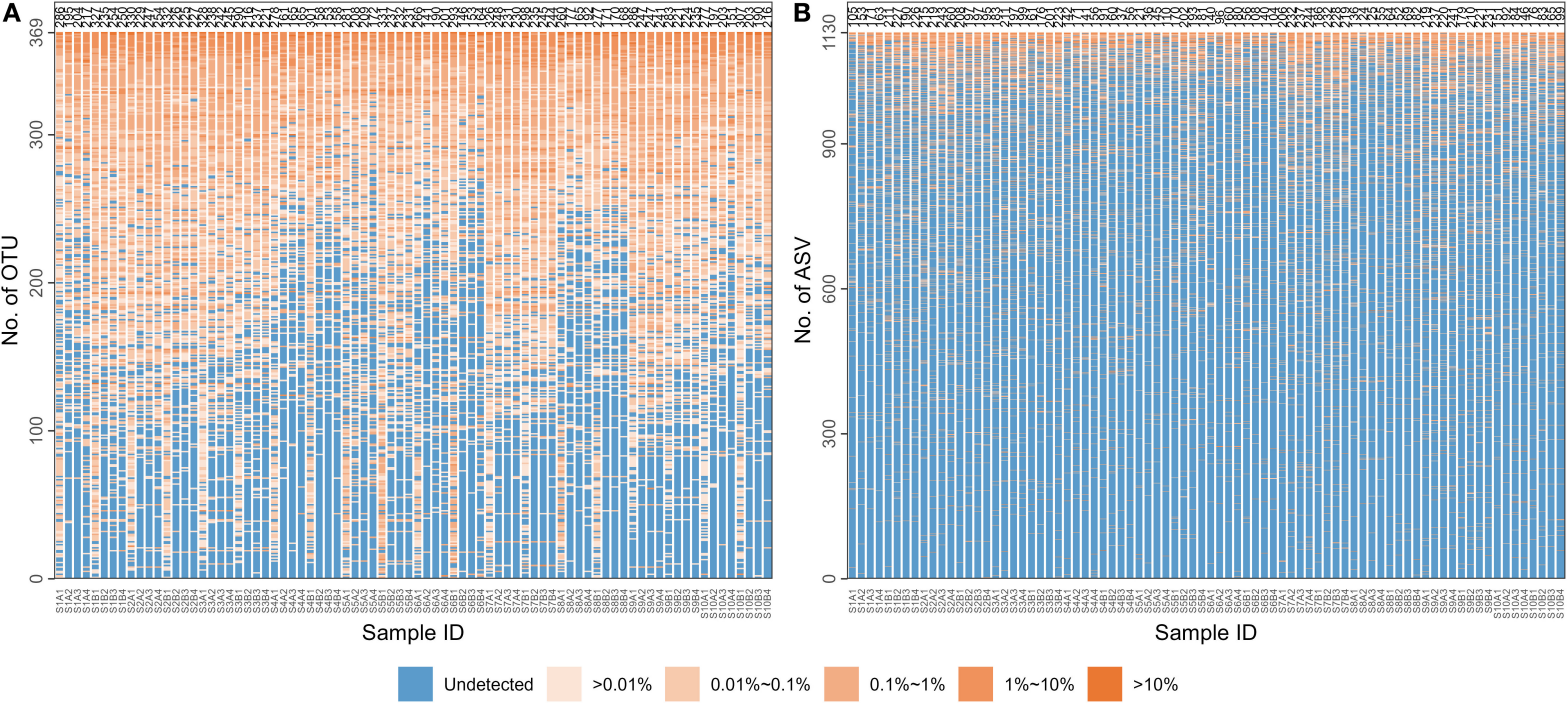
The distribution of microbial species characteristics in each sample. The microbiological information of 80 samples (each column for a sample) is represented at two levels: **(A)** Operational taxonomic unit (OTU); **(B)** Amplicon sequence variants (ASV). The numbers of OTUs or ASVs detected in each sample are listed at the top of each column. A color gradient represents the percentages for each detected OTU/ASV in the corresponding sample.

Supplementary Table 1 contains basic information about the 10 MZT pairs: age, gender, and population. After sequencing, 6,134,347 pairs of reads were obtained, with 6,107,044 clean reads remaining after splicing and filtering, as described in section 2.3. At least 48,389 clean reads were obtained for each of the 80 samples, with the mean number of clean reads per sample being 76,338. After clustering at 97% similarity and filtering with a threshold of 0.005% in total reads, 369 types of OTUs were detected across all 80 samples. The number of OTUs obtained per sample ranged from 141 to 332, averaging 230. As shown in Figure 1A, OTUs obtained in samples collected at the first time point for each participant would differ from the other three. Those differed significantly between inner-individual samples could be “burdens” on the identification model, implying the importance of feature selection. A total of 1130 ASVs were obtained after noise reduction using DADA2 and filtering with a 0.005% threshold in the 80 samples. Figure 1B indicates that the number of ASVs obtained per sample ranged from 96 to 260, with an average of 181.

**Figure 2:**
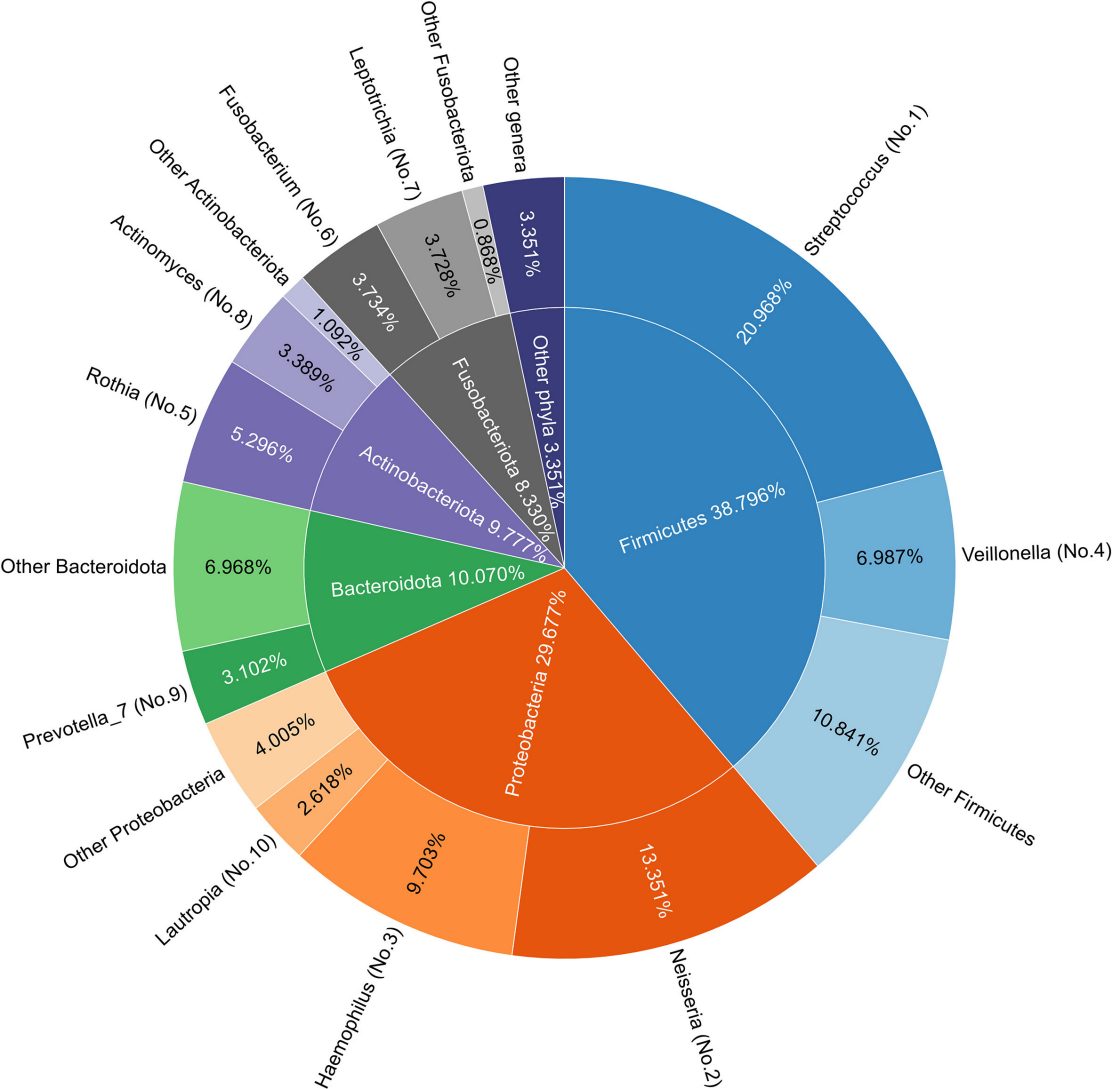
Species composition in all samples. **(Inner)** Pie chart of phylum composition of all samples: the top five phyla are arranged clockwise, with names and proportions marked in corresponding positions. **(Outer)** A doughnut chart of genus composition in all samples, with the top 10 genera listed. All genera belonging to the same phylum are grouped and labeled with a gradient of color for the corresponding phylum in the inner pie chart. The names and proportions of each genus are marked in the corresponding positions, where serial numbers in parentheses indicate the proportion rankings. Streptococcus and Neisseria are the most common genera in saliva and the top five genera accounting for more than half of all species.

Taxonomic annotation was performed using the SILVA 138 database, as mentioned in Section 2.3. There were 13 phyla detected in all 80 samples, with the top five (Firmicutes, Proteobacteria, Bacteroidota, Actinobacteriota, and Fusobacteriota) accounting for 97%. At the genus level, 131 genera were detected, with Streptococcus and Neisseria accounting for about 21 and 13% of the bacteria in saliva, respectively. The top five genera account for more than half of all species. Figure 2 depicts the taxonomic composition of all samples at the phylum and genus levels, and Supplementary Figure 1 indicates the compositions in each sample. Differences in taxonomic compositions can be found between both Self sample pairs and MZT sample pairs, indicating that microbiota information may have the potential to distinguish MZT. However, feature selection would be required in the model construction.

Basic information of OTUs and ASVs applied in the model construction is presented in Supplementary Table 2, including taxonomic annotation, Reads in each sample and the status of selection in the final model.

### 3.2. Preliminary analysis

#### 3.2.1. Alpha diversity analysis

**Figure 3:**
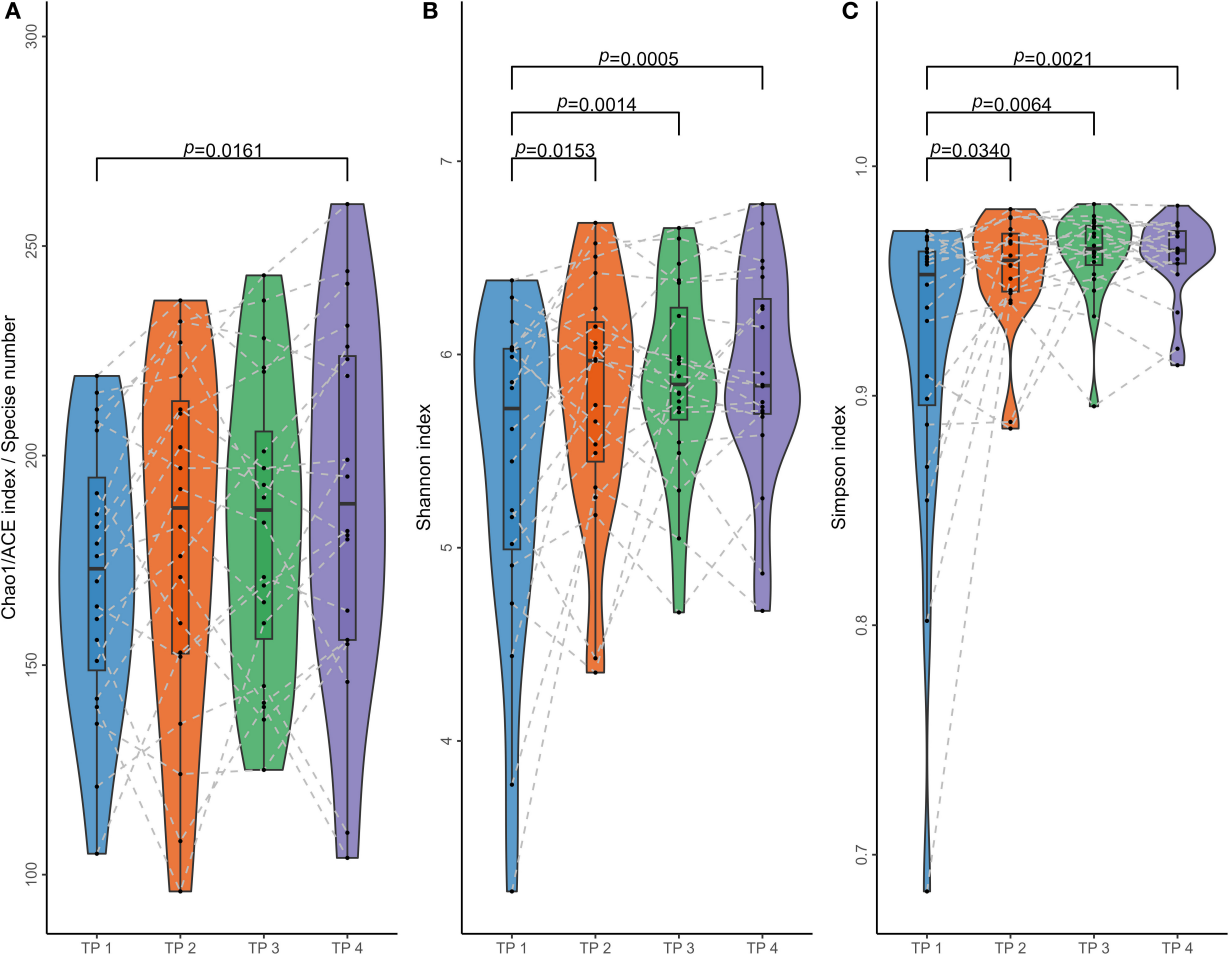
Paired comparisons of microbial alpha diversity in samples collected at different times. Four parameters were calculated to access microbial alpha diversity within a single sample and compare them in different time groups. **(A)** Chao 1 and ACE indices, measuring the species richness of a sample; **(B)** the Shannon indices and **(C)** Simpson indices, considering community evenness. These alpha diversity parameters were calculated using ASV sequence data of 80 samples from 20 individuals at four-time points (TP 1–4), with samples from TP 2/3/4 collected at least 1 year after TP 1. Dots present the parameters of each sample, and dotted lines link samples of the same individual. Paired *t*-test or Wilcoxon sign rank-sum test was performed for each of the six comparisons between the four groups, and *P* < 0.05 are listed in the figure.

Based on ASV information, four alpha diversity indices were calculated for each sample. After calculation, it was found that both ACE and Chao 1 indices of any sample equaled the number of ASV detected from the corresponding sample. For each individual, the four samples collected at different time points can form six comparisons; and for each type of comparison, paired *t*-test or Wilcoxon sign rank-sum test was performed to assess the difference between the distributions of the alpha indices between the corresponding two time points, according to whether the differences between all samples fit the normal distribution. As shown in Figure 3 and Table 2, if the time interval was <2 months within the same individuals, no significant difference could be found regardless of which index was calculated. More significant differences could be found between samples collected at the first time point and the other three if Shannon and Simpson's indices were more calculated than the other two.

**Table 2:** Comparison between α diversity between inner-individual samples.

**Parameter**	**Time points**	P1^*^	P2^**^	Note^***^
ACE/Chao 1	TP 1 vs. TP 2	0.1412	0.1332	T
	TP 1 vs. TP 3	0.8144	0.0675	T
	TP 1 vs. TP 4	0.4232	0.0161	T
	TP 2 vs. TP 3	0.7515	0.6714	T
	TP 2 vs. TP 4	0.6478	0.2619	T
	TP 3 vs. TP 4	0.0833	0.3042	T
Shannon	TP 1 vs. TP 2	0.0001	0.0153	R
	TP 1 vs. TP 3	0.0011	0.0014	R
	TP 1 vs. TP 4	0.0003	0.0005	R
	TP 2 vs. TP 3	0.0023	0.4221	R
	TP 2 vs. TP 4	0.0880	0.2026	T
	TP 3 vs. TP 4	0.8462	0.7192	T
Simpson	TP 1 vs. TP 2	0.2056	0.0340	T
	TP 1 vs. TP 3	0.1974	0.0064	T
	TP 1 vs. TP 4	0.3124	0.0021	T
	TP 2 vs. TP 3	0.0119	0.9854	R
	TP 2 vs. TP 4	0.2653	0.2760	T
	TP 3 vs. TP 4	0.5051	0.6319	T

* *P*-value of Shapiro-wilk tests on the differences of specific parameters in samples from the same individual.

** *P*-value of paired *t*-test (if corresponding *P*_1_>0.05) or Wilcoxon sign rank-sum test (if not) of each paired comparison, in which the significant differences (*P*_2_ < 0.05) are colored red and underlined.

*** T, paired *t*-test; R, Wilcoxon sign rank-sum test.

#### 3.2.2. PCoA based on beta diversity analysis

**Figure 4:**
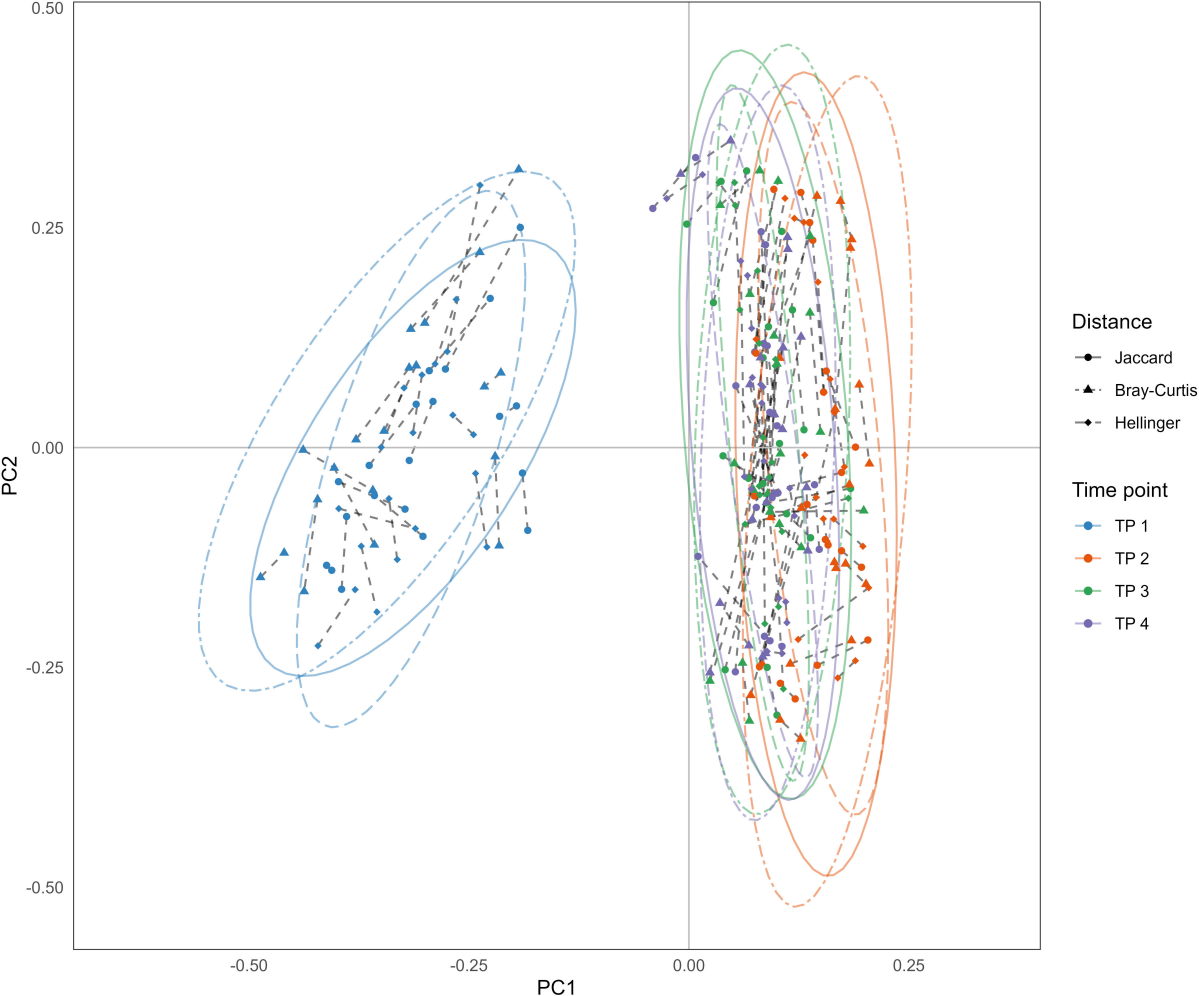
Results of principal coordinate analysis (PCoA). PCoA was performed using three types of beta density parameters: (i) Dots: Jaccard distance; (ii) Triangles: Bray-Curitis distance; (iii) Rhombus: Hellinger distance. Each point represents a sample, which is colored according to the time point of sample collection. Dotted lines connect samples collected from an MZT pair at the same time.

The three types of beta diversity parameters mentioned in Section 2.4.2 were used to perform principal coordinate analysis as shown in Figure 4, indicating that the results were similar to each other. The samples collected at the first time point were significantly different from other samples, i.e., the difference between the collection times point would overcome the difference between MZT pairs.

### 3.3. Feature selection with GA processes

As mentioned in Section 2.4.1, the 80 samples could form 280 Self or MZT sample pairs and be divided into four groups based on the relationship of the sourcing individuals and the time interval. Each group was randomly divided into training and test sets in an 8:2 ratio. A total of 12 GA processes were performed based on different feature-distance combinations in the training set, with the best AUC of each population generation listed in Supplementary Figure 2. Except for two GAs that stopped due to satisfying AUC = 1, the generational-best AUC of the remaining 10 GAs fluctuated within a relatively flat range after 1,000-1,500 iterations. The final output result can be considered close to the optimal solutions. Table 3 indicates the best AUCs for each GA, each of which is better than the corresponding AUC if all species detected were considered. Supplementary Figure 3 depicts the distance distributions based on the corresponding selected species and the KDE results. If the time interval is short and JD is used, the two groups could be completely separated, regardless of the choice in OTUs or ASVs. However, the KDE results indicate that if the final chosen combination of ASVs is used, the gap between the two curves would be larger, implying a better distinguishing ability. Meanwhile, when the time interval is long, the ASV-JD combination can also provide the best AUC. Therefore, ASV-JD combinations are selected for further model construction. As mentioned in Section 3.1, the status of whether each ASV is applied in the final model is annotated in Supplementary Table 2B.

**Table 3:** Best AUC achieved by 12 GA processes.

**Time interval**	**Species-level**	**Distance**	AUC_ALL^*^	Best AUC^**^	N^***^
Short	OTUs	Jaccard	0.6730	1	113
		Bray-Curtis	0.7299	0.8977	176
		Hellinger	0.7669	0.9560	134
	ASVs	Jaccard	0.8964	1	516
		Bray-Curtis	0.8448	0.9820	514
		Hellinger	0.8828	0.9880	514
Long	OTUs	Jaccard	0.5855	0.9123	111
		Bray-Curtis	0.6263	0.8320	141
		Hellinger	0.6385	0.8533	137
	ASVs	Jaccard	0.6795	0.9985	510
		Bray-Curtis	0.6102	0.9201	517
		Hellinger	0.6302	0.9536	494

* AUC in the training set if all detected features were involved in the model.

** Best AUC achieved after GA processes, the largest of which under each time interval are labeled red and underlined.

*** Number of features selected in the final model.

### 3.4. Construction and validation of the final models

**Figure 5:**
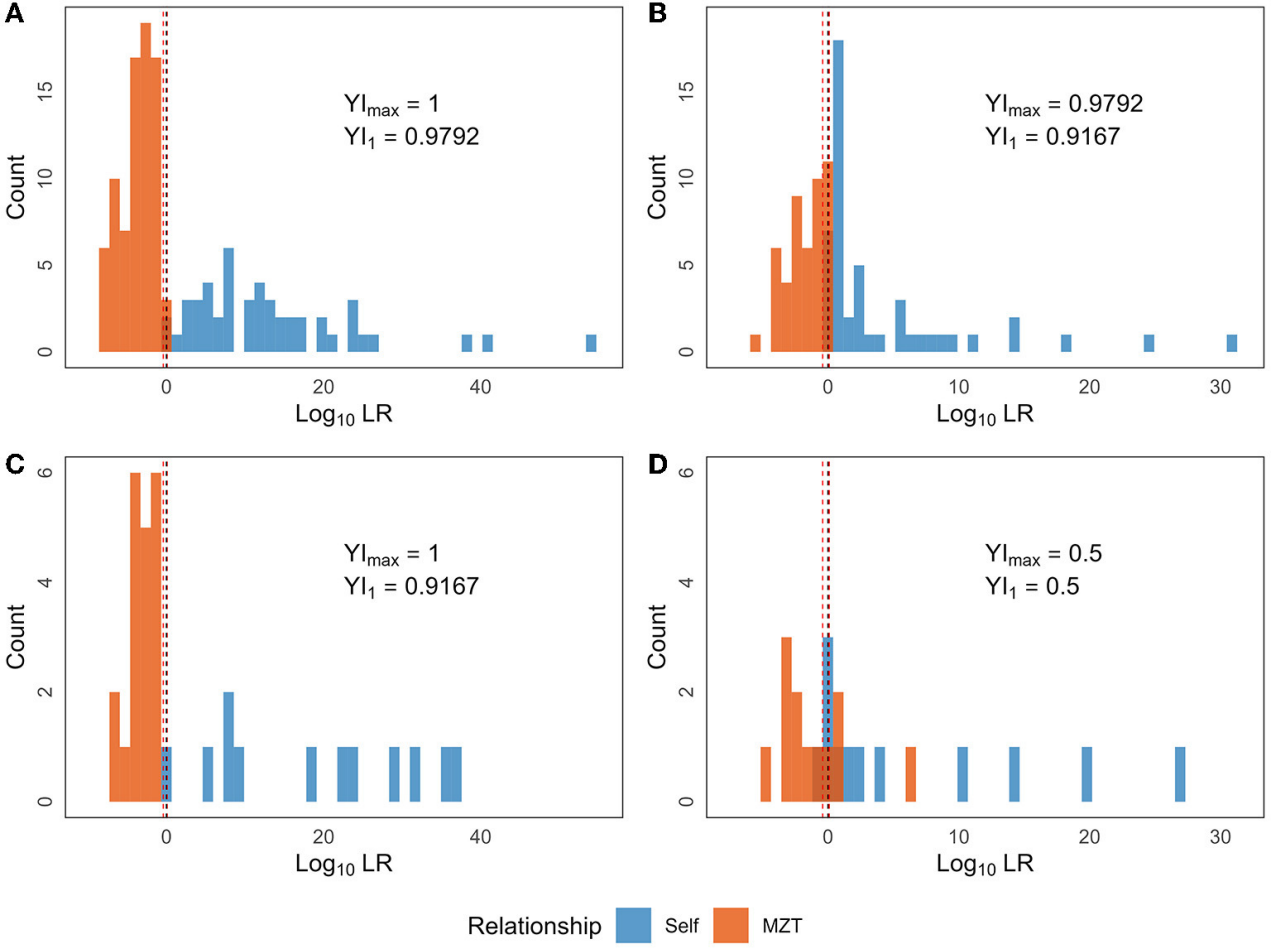
The LR distribution based on the final selected combination of ASVs. LR was calculated for each of the 280 sample pairs, which can be divided into eight groups based on the following three dimensions: (i) time interval (Long or Short); (ii) sourcing individual relationship (Self or MZT pair); (iii) training set or test set. The four sub-figures contain the LR distribution of Self (blue) or MZT (red) pairs in **(A)** Short+training set; **(B)** Long+training set; **(C)** Short+test set; **(D)** Long+test set. The red and black dotted lines denote *T*_*max*_ and LR = 1, respectively.

In the present study, the training set comprised 224 sample pairs. JD was calculated for each sample pair based on one of the two ASV combinations, as selected in Section 3.3, according to the time interval between the samples. Further, probability density curves were obtained for the four groups by processing the calculation results using KDE. These groups were segregated based on the relationship and time interval between the samples. Then, LR was computed for each of the 224 sample pairs and the corresponding results are shown in Table 4 (the first 4 rows) and Figures 5A, B. In the training set, the minimum LR of the Short-Self group was 0.8152, which was slightly higher than the maximum LR of Short-MZT group (0.3638). Therefore, if the confirming Self pair threshold is set between the two LR values (e.g., 0.4), the two groups in the training set can be completely separated, i.e., YI = 1. If LR = 1 is set as the threshold, one in 48 Short-Self pairs will be identified as an MZT pair, while no MZT pair will be mistakenly identified, i.e., YI = 0.9792. Meanwhile, in the training set, the minimum LR of the Long-Self group was 0.9553, and 3 Long-MZT pairs had LR higher than that. The best YI was achieved when the threshold was set between 1.2214 and 1.2548 when the Long-Self pair with the minimum LR would be the only pair incorrectly identified, and YI = 0.9792. If LR = 1 is set as the threshold, three Long-MZT pairs and one Long-Self pair would be mistakenly identified, resulting in YI = 0.9167.

**Table 4:** Model evaluation results.

**Data set**	**Time interval**	**Threshold**	**Sen**	**Spe**	YI^*^	**Misjudged pairs**
Training set	Short	$T_{max}=0.4^{**}$	1	1	1	None
		1	0.9792	1	0.9792	1 Self
	Long	*T*_*max*_ = 1.25	0.9792	1	0.9792	1 Self
		1	0.9792	0.9375	0.9167	1 Self and 3 MZT
Test set	Short	*T*_*max*_ = 0.4	1	1	1	None
		1	0.9167	1	0.9167	1 Self
	Long	*T*_*max*_ = 1.25	0.75	0.75	0.5	3 Self and 3 MZT
		1	0.75	0.75	0.5	3 Self and 3 MZT

* Sen, Sensitivity; Spe, Specificity; YI, Youden index; The calculation methods of these three parameters are presented in Equation (12).

** *T*_*max*_: the LR thresholds could provide the best YI in the training sets.

Furthermore, JD was calculated similarly for each sample pair in the test set, and then LR was calculated based on the above mentioned probability density curves. The two types of threshold sets were validated in the test set, as shown in Table 4 (the last four rows) and Figures 5C, D (the two lower sub-figures). Comparing to the training set, the model under short intervals could provide a similar YI in the corresponding test set. In contrast, two types of YI would drop sharply for identification between Long-Self and Long-MZT groups, from ~0.9 in training set to 0.5 (with both sensitivity and specificity being 0.75) in test set.

## 4. Discussion

In the present study, MZT identification models were built using microbiota 16s rRNA V3–V4 region information from 80 saliva samples collected from 10 pairs of MZT individuals while considering the time interval of sample collections. GAs were used to select high-value species-distance combinations for such models. As a result, JD calculated from 516 selected ASVs was used to construct a model that could completely separate MZT sample pairs collected in a short time interval ( ≤ 2 months, i.e., Short-MZT pairs) from Short-Self pairs in both the training and test sets. The distinguishing power of such a model was improved when compared to all 1,130 detected types of ASVs with the same distance parameter. A similar improvement in the GA process could be found for sample pairs with long time intervals (≥1 year), and 510 ASVs were selected to construct a model that would make correct decisions for 95 out of 96 pairs of the training set. However, the accuracy of such a model would drop sharply in the test set, where 6 of 24 pairs were misjudged, indicating the probability of over-fitting.

It is imperative that applied biomarkers exhibit individual specificity (i.e., distinction between different individual) to distinguish between MZT pairs effectively. Microbiota information is a more promising avenue for this purpose than the traditional genomic biomarkers, such as short tandem repeats (STRs) or single nucleotide polymorphisms (SNPs), which theoretically should be identical between MZTs. The first phase of the Human Microbiome Project (HMP) demonstrated that each collected sample contained a unique set of microorganisms (Human Microbiome Project Consortium, 2012), and multiple studies have highlighted the occurrence of similar differences between MZTs (Bowyer et al., 2022; Sukumar et al., 2023). The composition of an individual's microbiota undergoes a succession process throughout their lifetime (Martino et al., 2022), which can be influenced by several factors such as dietary habits, antibiotic treatments (Bokulich et al., 2016), diseases, or stochastic factors (Turnbaugh et al., 2010). While these factors shape the individual specificity of microbiota communities, they also continuously affect the stability of the communities. And another important requirement of the MZT distinguishing biomarkers is inner-individual stability, which ensures that the difference between the corresponding biomarkers from inter-individual samples is highly likely to exceed the difference between inner-individual samples. The sensitivity of different microorganisms to the above-mentioned factors should differ; the more sensitive species would significantly affect the inner-individual stability and thus “drag down” the application value of the MZT identification models. This could partially explain the improved performance of the model after the feature selection process, as represented by AUC (as illustrated in Table 3).

Based on ASV data, four types of alpha diversity parameters were calculated for each of the 80 samples. ACE and Chao 1 estimators measure species richness among these parameters, while Shannon and Simpson's indices consider community evenness. Analysis of Figure 3 reveals no significant differences in inner-individual samples collected at TP 2–4. Meanwhile, significant differences were identified between these samples and those collected at TP 1 when Shannon and Simpson's indices were used. This suggests that microbiota composition can be stable over a relatively short period of time. In contrast, calculating the ACE or Chao 1 estimator only revealed a significant difference between samples with the longest time intervals (14 months between TP 1 and TP 4). This indicates that the inner-individual stability of microbial species richness is superior to the stability considering community composition. This finding may help to explain why subsequent model constructions based on JD (which also considers only species richness) performed better under the same conditions than models based on the other two types of distances (which consider the community composition).

The calculated ACE and Chao 1 indices for each sample equaled the detected ASV of the corresponding sample. The reason would be that after being confirmed as ASVs, sequences were filtered with a 0.005% threshold, making the minimum reads of the final used ASVs > 48,389 × 0.005% = 2.41945. Consequently, parameters “F1” and “F2” used in the calculation of ACE and Chao 1 indices were set to zero for each sample, and the two indices equaled the observed ASV number, as shown in Equation (13).


(13) $$\{\begin{array}{ll} ACE & =S_{abund}+\frac{S_{rare}}{1-\frac{F1}{C_{ACE}}}+\frac{F1}{C_{ACE}}\gamma_{ACE}^{2}=S_{abund}+S_{rare}=S_{obs}\\ Chao\;1 & =S_{obs}+\frac{F1\left(F1-1\right)}{2\left(F2+1\right)}=S_{obs} \end{array}$$

Where *S*_*obs*_, *S*_*abund*_, and *S*_*rare*_ denote the total number of observed ASVs, the number of ASVs with large reads, and the number of ASVs with small reads; *F*1 and *F*2 represent the number of ASVs with reads 1 and 2, respectively. *C*_*ACE*_ and $\gamma_{ACE}^{2}$ can be calculated with several additional steps, but their values do not affect the final result when *F*1 = 0.

Based on the data presented in Supplementary Figure 3, it appears that distances exhibited a unimodal distribution across all 24 groups, accounting for three types of distances, two types of time intervals, two types of databases, and two types of sourcing individuals' relationships. Therefore, the conversion of LRs based on the aforementioned distributions results in a rough negative correlation with distance results. Consequently, there was no significant change in the numerical ranking of calculation results between samples, indicating that adding LR did not improve the distinguishing capability of the specific model in this study. Nevertheless, LR can be an effective tool for evaluating evidence credibility with respect to specific distance calculations. It enables a numerical representation of our confidence level in making a particular decision when faced with definite distance outcomes in real-world situations.

Limitations can be found in the present study. For example, unrelated sample pairs from different MZT pairs were not considered during the model construction process in this study. Fresh saliva samples were used, from which the host's DNA should be easily obtained. Therefore, individual identification issues other than MZT cases can be solved using traditional host-genome-level biomarkers like STRs or SNPs. If it is difficult to obtain the host's DNA, the sample is likely collected from another part of the body or is not fresh. The reference significance of results from fresh saliva samples to such cases would be reduced. This also highlights a problem that hinders using microorganisms in individual identification. The composition of microbial communities is greatly affected by the source body sites (Ward et al., 2018) and the time of isolation (Liu et al., 2022).

Another challenge to applying microbial 16s rRNA data in the forensic field is the lack of a standardized method of sequencing nomenclature, i.e., specific generic names for detected sequences, similar to the RefSNP (rs) system of SNP markers (Sherry et al., 2001). Because of this, the ASVs or OTUs in all samples were numbered sequentially, e.g., ASV1, and ASV2, which is obviously of a limited reference value. Such a problem would be covered if the model used all of the sequencing information, but it would be widened when feature selection is used. Before establishing the standardized method, researchers must construct their own microbial MZT identification models based on their sequencing data, instead of applying existing models, such as the ones we constructed in the present study.

Moreover, the present study used a relatively small sample size, which limited the consideration of various factors that could affect the model. For example, recent studies have demonstrated that co-habitation may be the primary factor in bacterial sharing between MZT pairs due to person-to-person oral microbiome transmission between co-living family members (Valles-Colomer et al., 2023). Therefore, there would be an increase in microbiota similarity between MZT pairs with increased co-habitation time, and a decrease after they live apart (Stahringer et al., 2012; Valles-Colomer et al., 2023). The number of co-living and separated pairs among the 10 MZT pairs investigated in the present study were four and six, respectively, which are insufficient for statistical analysis when considering time intervals and dividing data sets. Similar insufficiency would occur when considering gender, age, diseases, and antibiotics applications. Therefore, large-scale research and experimentation considering co-living factors may provide a more accurate assessment of the potential of microbiota information in resolving the MZT identification problem.

In summary, the investigation of the microbial solution to the MZT identification problem is still in its early stages. And thus, rather than recommending a preconceived model, the primary objective of the current research is to underscore the potential utility of feature selection in facilitating the process of model construction when identifying MZT pairs with microbial information.

## 5. Conclusions

The present study used 80 saliva samples from 10 pairs of MZT to develop models for identifying MZT based on their microbiota information. The feature selection method was used to build models that effectively distinguish MZT from Self pairs. When the sampling interval was <2 months, the final model could completely separate the two types of sample pairs. This highlights the potential value of microbiota information as a biomarker in MZT identification. Moreover, the feature selection process demonstrated its ability to refine models and filter out irrelevant data in this field, resulting in more accurate and reliable models.

## Data availability statement

The datasets presented in this study can be found in online repositories. The names of the repository/repositories and accession number(s) can be found at: NCBI—PRJNA983501.

## Ethics statement

The studies involving human participants were reviewed and approved by Medical Ethics Committee of Hebei Medical University. Written informed consent to participate in this study was provided by the participants' legal guardian/next of kin.

## Author contributions

SL, BC, and GF contributed to the conception and design of the study. GF, SD, QW, and XZ collected saliva samples. GF and SD performed 16s rRNA sequencing. GM conducted data analysis, carried out genetic algorithm in model construction, and wrote the first draft of the manuscript. LF and CL verified the model. BC and SL supervised the entire study and provided funding support. All authors have read, commented on, and approved the submitted version.
