# Supplementary material for: Feature selection with a genetic algorithm can help improve the distinguishing power of microbiota information in monozygotic twins' identification
Source: Front Microbiol. 2023 Jul 24;14:1210638. doi: 10.3389/fmicb.2023.1210638 (PMC10406218; doi:10.3389/fmicb.2023.1210638)
Supplement: Supplementary file 1 [file Data_Sheet_1.zip › Data Sheet 1.PDF]

Supplementary Material

1 SUPPLEMENTARY TABLES AND FIGURES

1.1 Figures

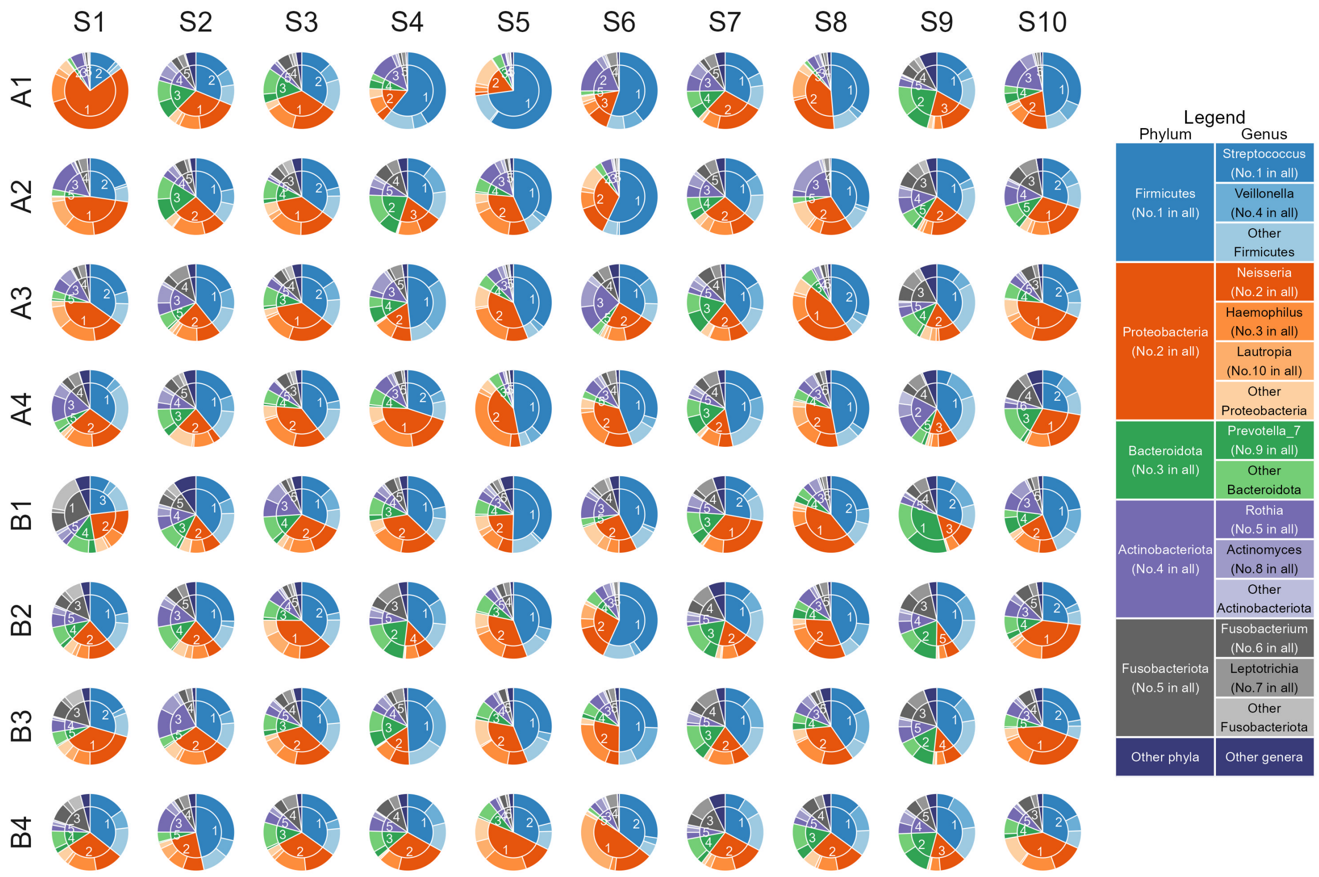

Figure S1. Species composition in each sample. The numbers of MZT pairs are listed on the top of the 80 subfigures, and the individual-time point labels are on the left. For each sample, the top 5 phyla are labeled in the inner pie chart.

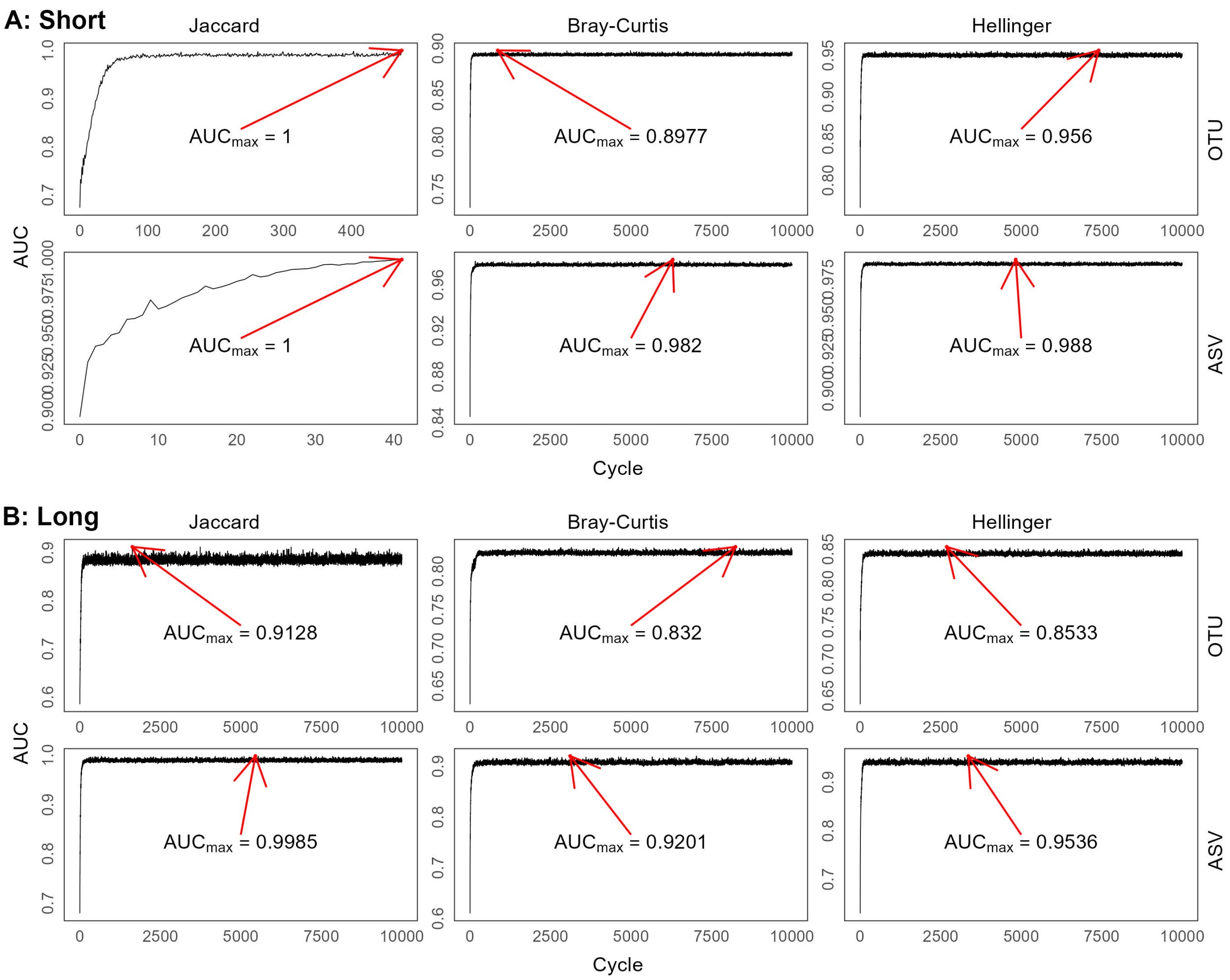

Figure S2. AUC change of each GA process. The best AUC of each GA process and the corresponding cycle is labeled in each subfigure.

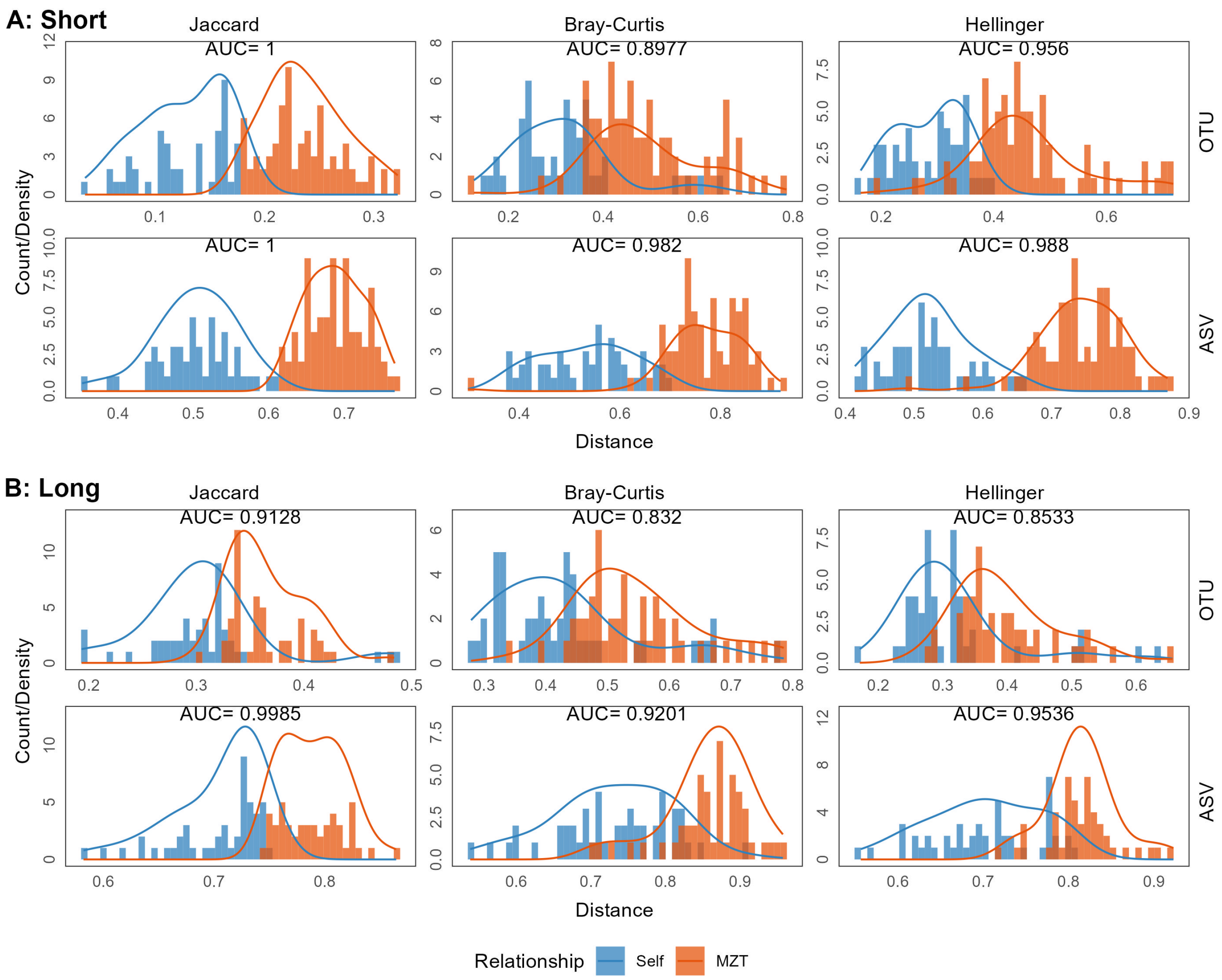

Figure S3. Distance distribution of the 12 best model provided by GA processes. The KDE results for each group are shown as lines in each subfigure.

1.2 Tables

Table S1. Basic information of the 10 MZT pairs

Number of pair	Number of individual	Age	Gender	Population
S1	S1A / S1B	41	Male	Han
S2	S2A / S2B	27	Female	Han
S3	S3A / S3B	38	Female	Han
S4	S4A / S4B	7	Female	Han
S5	S5A / S5B	26	Female	Han
S6	S6A / S6B	10	Female	Han
S7	S7A / S7B	29	Male	Han
S8	S8A / S8B	16	Male	Han
S9	S9A / S9B	46	Male	Han

Table S2. Basic information of OTUs and ASVs applied in the model construction

This is a large table presented in a .xlsx file.
